# Association of rs3027178 polymorphism in the circadian clock gene *PER1* with susceptibility to Alzheimer’s disease and longevity in an Italian population

**DOI:** 10.1007/s11357-021-00477-0

**Published:** 2021-12-18

**Authors:** Maria Giulia Bacalini, Flavia Palombo, Paolo Garagnani, Cristina Giuliani, Claudio Fiorini, Leonardo Caporali, Michelangelo Stanzani Maserati, Sabina Capellari, Martina Romagnoli, Sara De Fanti, Luisa Benussi, Giuliano Binetti, Roberta Ghidoni, Daniela Galimberti, Elio Scarpini, Marina Arcaro, Enrica Bonanni, Gabriele Siciliano, Michelangelo Maestri, Biancamaria Guarnieri, Italian Multicentric Group on clock genes, actigraphy in AD, Federico Cucchiara, Alessandro Schirru, Annalisa Lo Gerfo, Gemma Lombardi, Dario Arnaldi, Pietro Mattioli, Flavio Nobili, Gianluigi Cerroni, Antonella Bartoli, Raffaele Manni, Elena Sinforiani, Michele Terzaghi, Maria Grazia Arena, Rosalia Silvestri, Maria Caterina Di Perri, Ferdinando Franzoni, Gloria Tognoni, Michelangelo Mancuso, Sandro Sorbi, Ubaldo Bonuccelli, Ugo Faraguna, Morena Martucci, Daniela Monti, Valerio Carelli, Claudio Franceschi, Chiara La Morgia, Aurelia Santoro

**Affiliations:** 1 IRCCS Istituto delle Scienze Neurologiche di Bologna, Laboratorio Brain Aging, Bologna, Italy; 2 IRCCS Istituto delle Scienze Neurologiche di Bologna, Programma di Neurogenetica, Bologna, Italy; 3 Department of Experimental, Diagnostic and Specialty Medicine, University of Bologna, Bologna, Italy; 4 Applied Biomedical Research Center (CRBA), S. Orsola-Malpighi Polyclinic, Bologna, Italy; 5 CNR Institute of Molecular Genetics “Luigi Luca Cavalli-Sforza”, Unit of Bologna, Bologna, Italy; 6 Department of Laboratory Medicine, Clinical Chemistry, Karolinska Institutet, Karolinska University Hospital, Stockholm, Sweden; 7 Alma Mater Research Institute on Global Challenges and Climate Change (Alma Climate), University of Bologna, Bologna, Italy; 8 Laboratory of Molecular Anthropology and Centre for Genome Biology, Department of Biological, Geological and Environmental Sciences, University of Bologna, Bologna, Italy; 9 IRCCS Istituto delle Scienze Neurologiche di Bologna, UOC Clinica Neurologica, Bologna, Italy; 10 Department of Biomedical and Neuromotor Sciences (DIBINEM), University of Bologna, Bologna, Italy; 11 IRCCS Istituto Centro San Giovanni di Dio Fatebenefratelli, Brescia, Italy; 12 Fondazione IRCCS Ca’ Granda, Ospedale Policlinico, Milan, Italy; 13 Dino Ferrari Center, University of Milan, Milan, Italy; 14 Neurology Unit, Department of Clinical and Experimental Medicine, University of Pisa, Pisa, Italy; 15 Center of Sleep Medicine, Villa Serena Hospital and Villaserena Foundation for the Research, Città S. Angelo, Pescara, Italy; 16 Department of Experimental and Clinical Biomedical Sciences “Mario Serio”, University of Florence, Florence, Italy; 17 Department of Applied Mathematics, Institute of Information Technology, Mathematics and Mechanics (ITMM), Lobachevsky State University of Nizhny Novgorod-National Research University (UNN), Nizhny Novgorod, Russia

**Keywords:** Aging, Alzheimer’s disease, Centenarians, CLOCK genes, Polymorphism, Circadian rhythms

## Abstract

Many physiological processes in the human body follow a 24-h circadian rhythm controlled by the circadian clock system. Light, sensed by retina, is the predominant “zeitgeber” able to synchronize the circadian rhythms to the light-dark cycles. Circadian rhythm dysfunction and sleep disorders have been associated with aging and neurodegenerative diseases including mild cognitive impairment (MCI) and Alzheimer’s disease (AD). In the present study, we aimed at investigating the genetic variability of clock genes in AD patients compared to healthy controls from Italy. We also included a group of Italian centenarians, considered as super-controls in association studies given their extreme phenotype of successful aging. We analyzed the exon sequences of eighty-four genes related to circadian rhythms, and the most significant variants identified in this first discovery phase were further assessed in a larger independent cohort of AD patients by matrix assisted laser desorption/ionization-time of flight mass spectrometry. The results identified a significant association between the rs3027178 polymorphism in the *PER1* circadian gene with AD, the G allele being protective for AD. Interestingly, rs3027178 showed similar genotypic frequencies among AD patients and centenarians. These results collectively underline the relevance of circadian dysfunction in the predisposition to AD and contribute to the discussion on the role of the relationship between the genetics of age-related diseases and of longevity.

**Supplementary Information:**

The online version contains supplementary material available at 10.1007/s11357-021-00477-0.

## Introduction

The circadian clock is an evolutionary-conserved internal time-keeping system, able to control various physiological processes through the generation of approximately 24-h circadian rhythms in gene expression, which are translated into rhythms of metabolism, sleep, body temperature, blood pressure, cardiovascular, immune, endocrine and renal functions [1, 2]. Two major components include a central clock, residing in the suprachiasmatic nucleus (SCN) of the hypothalamus, and the peripheral clocks, present in nearly every tissue and organ system. Both central and peripheral clocks can be reset by environmental signals, also known as “zeitgebers”, the predominant of which for the central clock is light, sensed by retina and synchronizing the circadian rhythms to the light-dark cycles [3, 4]. The central clock entrains the peripheral ones through neuronal and hormonal signals, body temperature and feeding-related stimuli, ultimately aligning all clocks with the external light/dark cycle.

In mammals, the regulation of circadian oscillators occurs through a series of positive/negative transcriptional-translational feedback loops including at least nine core circadian genes [5]. Among them, period homolog (PER1, PER2 and PER3) and cryptochrome (CRY1 and CRY2) clock proteins form complexes to negatively inhibit the nuclear transcription activities of the heterodimers formed by the transcription factors circadian locomotor output cycles kaput (CLOCK) [6] with aryl hydrocarbon receptor nuclear translocator-like protein 1 (ARNTL; also known as BMAL1) [7, 8]. Circadian gene regulation is a complex, temporally orchestrated process that involves not only the main circadian factors mentioned above but also a growing list of secondary or cell type-specific transcription factors, transcription co-regulators and epigenetic activities [4].

The synchronization of the endogenously generated circadian clocks to the light-dark cycle is possible thanks to the projections of the retinal ganglion cells expressing the photopigment melanopsin (mRGCs) to the SCN through the retino-hypothalamic tract [9–11]. The mRGCs are a small subgroup of intrinsically photosensitive RGCs (about 1% of the total), particularly sensitive to blue light. They mediate circadian photo-synchronization and other “non-image forming” functions of the eye [9, 10, 12]. Single nucleotide polymorphisms (SNPs) in the opsin 4 (*OPN4*) gene encoding for the melanopsin photopigment have been associated with seasonal affective syndrome (SAD), pupillary response to light and season-related chronotype [13–17].

A large body of evidence supports an association between disruption of circadian rhythms and neurodegenerative diseases [18, 19]. Disruption of circadian rhythms and sleep disorders frequently occur in patients with Alzheimer’s disease (AD), showing reduced amplitude of circadian rhythms, increased sleepiness and fragmented sleep-wake patterns as compared to healthy individuals [20–23]. Poor circadian functioning has been associated to increased risk to develop mild cognitive impairment and dementia in older women [24], and there is increasing evidence that sleep disorders favor the accumulation of ß-amyloid in the brain [25] and that circadian dysfunction can have a negative impact on cognitive functions [23]. Importantly, the alterations in circadian rhythms observed in AD resemble and exacerbate those occurring during physiological aging [26–29], sustaining a link between age-related changes and neurodegenerative diseases [30]. Decrease in melatonin levels, reduction of the amplitude of peripheral oscillatory rhythms, changes in SCN network and gene expression have been described during aging (reviewed in [18]). Furthermore, in vivo studies with optical coherence tomography (OCT) and post mortem histological studies have shown age-related loss of RGCs including mRGCs [31–33]. Moreover, a specific loss of mRGCs as well as a deposition of amyloid in human AD retinas has been reported [33].

SNPs located in circadian clock genes have been associated with an extensive range of phenotypes and pathological conditions, including cancer, metabolic diseases and psychiatric disorders [34]. However, only a few studies have specifically considered their association with AD so far.

Thus, we investigated whether genetic polymorphisms in circadian clock genes, including *OPN4*, are associated with AD. Given the importance of aging in AD-associated circadian dysfunction, we included in our analysis not only healthy age-matched controls, but also a cohort of centenarians (CENT). Centenarians represent an extreme phenotype of successful aging [35], characterized by specific nutritional habits [36, 37], a peculiar gut microbiota [38, 39], a well-preserved sleep quality and quantity [36, 40] and a particular genetic background [41]. For these reasons, they can be considered a group of “super-controls” to gain information on the biological relevance of genetic risk factors for common age-related diseases [42].

The present study was conducted in two phases. In the discovery phase, the exon sequences of eighty-four genes related to circadian rhythms have been analyzed in a cohort of 79 AD and 33 mild cognitive impairment (MCI) patients compared to 62 controls (CTRL). Subsequently, in the validation phase, the most significant variants identified in the discovery phase were validated in a cohort of 449 AD patients, 326 CTRL and 152 CENT.

## Materials and methods

### Study population: discovery and validation cohorts

In this study, DNA samples from 1101 unrelated northern Italian subjects were analyzed. In the discovery phase, we included 79 AD, 33 MCI and 62 CTRL, recruited at the IRCCS Istituto delle Scienze Neurologiche di Bologna, Bellaria Hospital in the framework of an Italian multi-centric study and as part of a research project funded by the Italian Ministry of Health (GR- 2013- 02358026 to CLM and AS) [43]. In the validation phase, we analyzed an independent group including 449 AD, 326 age-matched healthy CTRL and 152 centenarians (CENT). The geographic origin and the number of samples for each participating group were the following: Bologna (77 CTRL and 152 CENT recruited at the Department of Experimental, Diagnostic and Specialty Medicine (DIMES) of the University of Bologna), Brescia (249 AD; 249 CTRL recruited at the MAC Memory Clinic, IRCCS Istituto Centro San Giovanni di Dio Fatebenefratelli), Milan (200 AD recruited at Fondazione IRCCS Ca’ Granda, Ospedale Policlinico). Age and sex distribution of discovery and validation cohorts are described in Table 1. Written informed consent was obtained from all control individuals and primary caregivers on behalf of AD patients. Each Institution that provided the DNA samples received the approval from their own ethical committees. In particular, the following Ethic committees gave their approval: IRCCS Bellaria Hospital, CE 16032 and Sant’Orsola-Malpighi University Hospital in Bologna (reference n°22/2007/U/Tess issued on 27/02/2007 and amendment n. EM 157/2011/U issued on 25/11/2011), IRCCS Istituto Centro San Giovanni di Dio Fatebenefratelli in Brescia (Approval number 92-2019, issued on the 04/12/2019), Fondazione IRCCS Ca’ Granda, Ospedale Policlinico in Milan (Approval number 532-2019, issued by the CE Milan Area2). DNA was extracted from whole blood in the different recruiting centers and plated for quality control and quantification.

**Table 1:** Characteristics of the studied cohorts

Cohort	All (*N*)	Males (*N*)	Females (*N*)	Mean age±SD
Discovery				
AD	79	37 (46.8%)	42 (53.2%)	75.6±9.5
MCI	33	18 (54.5%)	15 (45.5%)	80.0±7.1
CTRL	62	26 (41.9%)	36 (58.1%)	69±11.8
Validation				
AD	449	115 (25.6%)	334 (74.4%)	76.5 ± 6.9
CTRL	326	126 (38.6%)	200 (61.4%)	66.1 ± 7.2
CENT	152	33 (21.7%)	119 (78.3%)	102.5 ± 2.9

All subjects were of Italian origin. AD patients evaluated in the discovery phase were diagnosed by skilled clinical neurology units as suffering from probable AD, according to Dubois criteria [44], whereas for the validation phase NINDS-ADRDA criteria have been used for AD diagnosis [45]. The AD patients included for the discovery phase underwent a comprehensive neurological assessment, including an extended neuropsychological evaluation which included: for memory evaluation the Rey’s 15 Words (immediate recall and delayed recall), Immediate visual memory, Digit span (forward and backward), the Rey-Osterrieth complex figure test (ROCF) for delayed recall; for Attention the Barrage Test and the Stroop Test; for Language the Verbal fluency test (phonemic and semantic); for constructive praxis the Simple copy design, the Rey-Osterrieth complex figure test (ROCF) for direct copy; for Visuospatial and Perceptual functions the Corsi Span Task forward, the Judgment of line orientation test and the Street’s Completion Test; and for Abstract/concrete thinking-intelligence the Analogies test. MCI patients were enrolled according to Petersen criteria [46]. The control group was free of clinically evident major diseases and was assessed by Mini Mental State Examination (MMSE) test as well as the Brief Mental Deterioration Battery (BBDM) [47, 48] in order to include subjects not affected by cognitive deficiency (MMSE >27). The health status of centenarians was more heterogeneous than younger controls: while the majority was in good health, some suffered from multiple late-onset age-related diseases, intrinsic to their status of centenarians [49].

### Next-generation sequencing

A custom NGS panel with 84 genes related to circadian rhythms and melanopsin (Table s1) was based on a commercial kit (RT2 Profiler PCR Array, Qiagen) and designed with the Nextera DNA Flex Library Prep (Illumina Inc., San Diego, CA). Libraries were prepared from total blood’s DNA and were sequenced as 151-bp paired-end reads on NextSeq 500 platform (Illumina Inc., San Diego, CA). BCL files were demultiplexed and converted to the FASTQ format with the Illumina standalone bcl2fastq program (v2.20.0.422). Generated reads were aligned with BWA [50] to the reference genome hg19, realignment and base quality score recalibration were performed with GATK [51] and duplicate removal with PicardTools (https://broadinstitute.github.io/picard/). Alignment and coverage statistics were collected with SAM tools [52] and GATK. Variants were called and filtered by quality with GATK UnifiedGenotyper and VariantFiltration, then annotated with RefSeq using SnpEff [53].

### Case-control study and CMC analysis

A case-control study was performed on SNPs identified in the discovery phase and filtered with VCF tools [54]. Briefly, variants were filtered out if they were multi-allelic, non-PASS, with a variant call rate <95%, *singletons*, with a Hardy-Weinberg Equilibrium (HWE) test *p* value <10^−6^ and with minor allele frequency (MAF) ≤5% respect to the 1000 Genomes database. Allelic frequencies were compared in case and control through a Fisher’s exact test in PLINK v1.90 [55]. A nominal *p* value ≤0.01 was considered significant. Rare variant distribution within cases and controls was tested with a CMC (collapsing and combine) test as described elsewhere [56]*.* We defined qualifying variants as PASS variants with HIGH (stopgain, frameshift indels, canonical splicing) and MEDIUM (missense CADD>15) impact, with a MAF<1% in the ExAC database and never observed in the homozygous state in the GnomAD database. The null hypothesis of equality of proportions of *cases and controls* with at least one qualifying variant was tested with an exact unconditional test [57] in R3.6.0 using Package “exact2x2” (https://www.R-project.org/).

### Genotyping

Genotyping was performed using the iPLEX assay on MassArray system (Agena) on 449 AD patients, 326 CTRL and 152 CENT (validation phase). The Assay Design Suite (Agena) was used to design primers against 14 candidate SNPs selected from NGS analysis. PCR products were processed following the manufacturer’s instructions and analyzed by matrix-assisted laser desorption/ionization-time of flight (MALDI-TOF) mass spectrometry. The MassARRAY Typer 4.0 software was used to call genotypes, and the individual spectrograms were inspected to check for calling errors [58]. Statistical analysis was performed using PLINK v1.90. rs1134224 had a call rate <0.95 and was excluded from the analysis. No significant deviations from HWE (*p* value<0.001) were evident for the remaining SNPs. Twenty-five individuals (10 AD, 14 CTRL and 1 CENT) had a genotyping rate <0.75 and were excluded from the further analysis. Logistic regression was used to correlate SNPs with proband status, using sex as covariate.

### Functional analysis

GTEx portal (https://gtexportal.org/home/) was interrogated to investigate the association between SNPs and transcripts levels in different tissues. The normalized effect size (NES) indicates the effect of the alternative allele relative to the reference allele in the human genome reference, calculated for each tissue.

## Results

**Fig. 1:**
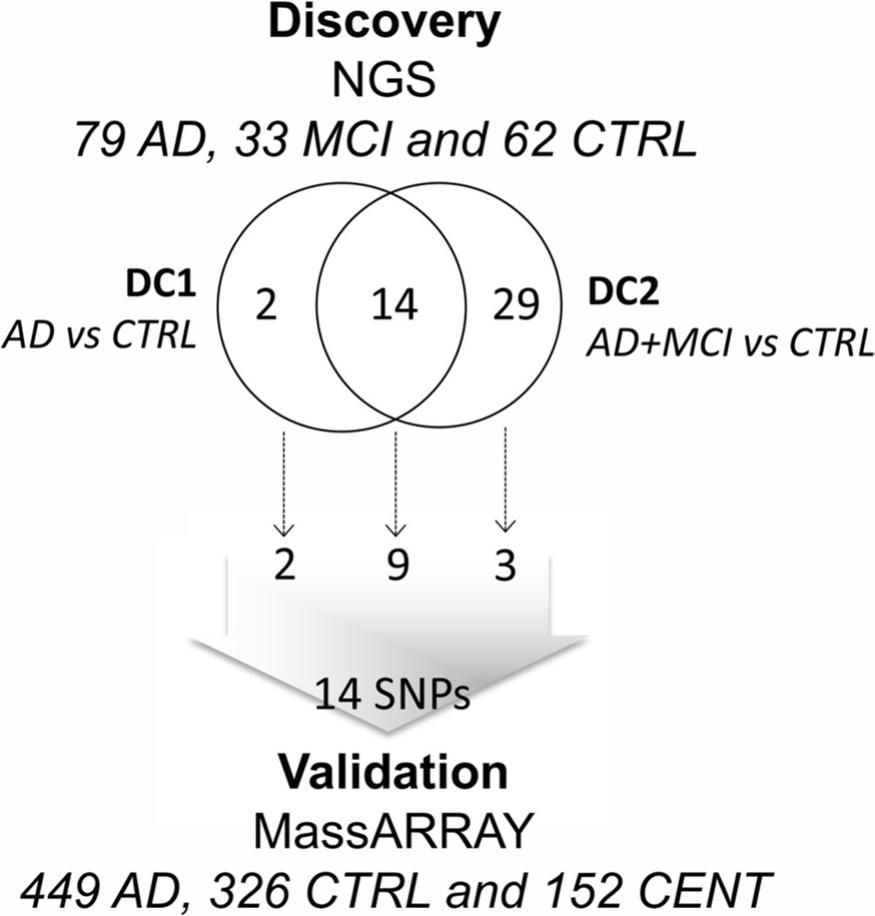
**Flow chart of the study.** The present study has been conducted in two phases. In the discovery phase, a NGS protocol was applied to study 84 genes related to circadian rhythms in a restricted cohort of AD and CTRL subjects (discovery cohort 1—DC1) and in a larger cohort including also MCI patients (discovery cohort 2—DC2). Sixteen and forty-three nominally significant variants were identified in DC1 and DC2 respectively, fourteen of which were in common. A selection of the variants identified in the discovery phase was then analyzed by a custom genotyping SNP array in a larger cohort of AD patients, CTRL and CENT (validation phase).

We used a two phases approach to investigate the genetic variability of clock and melanopsin genes in AD patients compared to controls and centenarians (Figure 1).

### Discovery phase

In the discovery phase, a NGS assay including the exon region of 84 selected genes related to circadian rhythms and melanopsin (see Table s1) was applied to a cohort including 79 AD, 33 MCI patients and 62 CTRL. The quality of the NGS assay was very high, with an average coverage of 986X (±336X) and 98% (±1%) of the bases covered at least at 20X.

The discovery cohort was further divided into two sub-cohorts, a discovery cohort 1 (DC1) including AD and CTRL (*N*=141 subjects, 79 AD and 62 CTRL) and a discovery cohort 2 (DC2) which also includes MCI patients among the cases (*N*=174 subjects, 79 AD, 33 MCI and 62 CTRL). After filtering steps (Figure s1), 753 and 752 SNPs were evaluated in the DC1 and DC2, respectively (File s1).

Sixteen and forty-three nominally significant SNPs were obtained in DC1 and DC2, respectively, 14 of which were in common (Table 2 and File s1).

**Table 2:** SNPs identified in the discovery phase, considering DC1 (AD vs CTRL) and DC2 (AD+MCI vs CTRL)

CHR	BP	dbSNP	A1	A2	DC1						DC2						Validation
					P	OR	L95	U95	F AD	F CTR	P	OR	L95	U95	F AD	F CTR	
17	8052525		C	CAAAAACA	0.000	0.416	0.257	0.673	0.405	0.621	0.001	2.107	1.343	3.305	0.554	0.371	
10	88414758	rs2254051	A	G	0.001	0.347	0.182	0.660	0.108	0.258	0.001	0.381	0.213	0.681	0.113	0.250	X
10	88421997	rs2675698	T	C	0.001	0.347	0.182	0.660	0.108	0.258	0.001	0.381	0.213	0.681	0.113	0.250	
10	88419290	rs2254548	A	C	0.002	0.362	0.189	0.691	0.108	0.250	0.004	0.416	0.233	0.742	0.117	0.242	
10	88414569	rs2675703	T	C	0.003	0.370	0.196	0.697	0.114	0.258	0.002	0.398	0.224	0.708	0.117	0.250	X
20	5283256	rs3746682	C	G	0.004	0.444	0.259	0.760	0.196	0.355	0.000	0.398	0.242	0.655	0.185	0.363	X
6	36075219	rs9470219	A	C	0.004	2.106	1.272	3.486	0.443	0.274	0.002	2.140	1.326	3.453	0.437	0.266	X
1	151780177	rs3828057	T	C	0.005	0.485	0.294	0.798	0.272	0.436	0.002	0.469	0.295	0.746	0.266	0.436	X
10	92617609	rs11186339	C	T	0.006	0.181	0.050	0.655	0.019	0.097	0.003	0.215	0.074	0.626	0.023	0.097	
6	36041431	rs851010	A	G	0.006	2.023	1.225	3.340	0.443	0.282	0.003	2.092	1.301	3.365	0.441	0.274	X
9	77286876	rs3818559	C	A	0.008	2.115	1.223	3.655	0.348	0.202	0.002	2.275	1.357	3.814	0.365	0.202	X
22	46633371	rs1134224	T	A	0.009	2.559	1.263	5.184	0.215	0.097	0.008	2.418	1.255	4.662	0.221	0.105	X
22	46633782	rs6008259	A	G	0.009	2.559	1.263	5.184	0.215	0.097	0.008	2.418	1.255	4.662	0.221	0.105	X
22	46638298	rs1055659	T	C	0.009	2.559	1.263	5.184	0.215	0.097	0.008	2.418	1.255	4.662	0.221	0.105	
20	5294496	rs8116897	T	C	0.005	2.032	1.253	3.297	0.519	0.347	ns						X
17	8053085	rs3027178	G	T	0.007	0.485	0.292	0.805	0.253	0.411	ns						X
4	56297874	rs35543551	C	CAG	ns						0.002	0.487	0.309	0.766	0.306	0.476	
4	56300685	rs3749474	T	C	ns						0.004	0.502	0.319	0.788	0.320	0.484	
4	56301049	rs5858333	GA	G	ns						0.004	0.502	0.319	0.788	0.320	0.484	
4	56297762	rs6828570	G	C	ns						0.004	0.512	0.326	0.803	0.324	0.484	X
4	56295873	rs62303689	A	C	ns						0.004	2.894	1.356	6.179	0.185	0.073	X
4	56295583	rs1047354	G	A	ns						0.005	0.518	0.330	0.814	0.320	0.476	X
17	38252667	rs939346	G	A	ns						0.006	0.000	0.000	*NA*	0.000	0.040	
4	56411741	rs7691799	A	C	ns						0.006	0.523	0.333	0.820	0.329	0.484	
1	241757409		TA	T	ns						0.007	2.278	1.260	4.118	0.266	0.137	
22	46636976	rs41378347	A	G	ns						0.007	2.900	1.305	6.446	0.167	0.065	
22	46637239	rs41427746	C	T	ns						0.007	2.900	1.305	6.446	0.167	0.065	
22	46637254	rs9626814	A	G	ns						0.007	2.900	1.305	6.446	0.167	0.065	
22	46638128	rs45550937	G	C	ns						0.007	2.900	1.305	6.446	0.167	0.065	
22	46638159	rs10154348	A	C	ns						0.007	2.900	1.305	6.446	0.167	0.065	
22	46638171	rs45552534	A	G	ns						0.007	2.900	1.305	6.446	0.167	0.065	
22	46638211		A	G	ns						0.007	2.900	1.305	6.446	0.167	0.065	
22	46638216	rs45576140	G	C	ns						0.007	2.900	1.305	6.446	0.167	0.065	
22	46638312	rs45528736	G	A	ns						0.007	2.900	1.305	6.446	0.167	0.065	
22	46638365	rs45576734	G	A	ns						0.007	2.900	1.305	6.446	0.167	0.065	
22	46638486		T	C	ns						0.007	2.900	1.305	6.446	0.167	0.065	
4	56329773	rs1522112	C	T	ns						0.008	0.529	0.337	0.830	0.324	0.476	
4	56355477	rs6838882	G	A	ns						0.008	0.529	0.337	0.830	0.324	0.476	
4	139965724	rs3805213	T	C	ns						0.008	0.476	0.280	0.810	0.158	0.282	
4	56296763	rs1056547	G	T	ns						0.008	0.533	0.340	0.836	0.333	0.484	
4	56296897	rs1056545	A	G	ns						0.008	0.533	0.340	0.836	0.333	0.484	
4	56296907	rs5863	G	A	ns						0.008	0.533	0.340	0.836	0.333	0.484	
4	56412169	rs12505266	T	C	ns						0.008	0.533	0.340	0.836	0.333	0.484	
4	56337041	rs3805151	C	T	ns						0.008	0.544	0.347	0.853	0.338	0.484	
9	77245348		C	CG	ns						0.009	0.436	0.234	0.811	0.099	0.202	

*CHR* chromosome, *BP* position (GrCH 37/hg19), *dbSNP* rs ID, *A1* minor allele, *A2* major allele, *P* nominal *p* value, *OR* odds ratio, *L95* lower bound of 95% confidence interval for odds ratio, *U95* upper bound of 95% confidence interval for odds ratio, *F AD* frequency in AD in DC1, or frequency in AD+MCI in DC2, *F CTR* frequency in control

Additionally, 32 rare qualifying variants were tested with the CMC method among cases and controls. The qualifying variants were distributed as follows: 18 in AD (22%), 19 in AD+MCI (17%) and 16 in CTRL (25%) (File s1). No evidence of enrichment of rare variants was found (*p* = 0.69 and *p* = 0.17, respectively).

### Validation phase

Fourteen of the SNPs identified in the discovery phase were selected to be validated by high-throughput genotyping assay based on the MALDI-TOF mass spectrometry technology (iPLEX assay), which was applied on a larger, independent cohort including 449 AD, 326 CTRL and 152 CENT. To this aim, we selected nine of the SNPs in common between DC1 and DC2, two SNPs exclusive for DC1 (mapping in *PER1* and *PROKR2* genes) and three SNPs exclusive for DC2 (mapping in *CLOCK* gene) (Tables 2 and s2). One SNP (rs1134224) and 25 subjects were excluded from the analyses after quality checks (see “Materials and methods”).

The comparison between AD and CTRL subjects by means of logistic regression corrected for sex revealed statistically significant differences only for the rs3027178 variant located in the *PER1* gene (nominal *p* value = 0.046), with the minor allele G resulting protective for AD (OR: 0.803; 95% confidence interval [CI]: 0.647–0.996) (Table 3). We then repeated the analysis combining the discovery (DC1, 79 AD and 62 CTRL) and validation cohorts, for a total of 528 AD and 388 CTRL (Table 4). The association of rs3027178 was confirmed and was statistically significant also after correction for multiple testing (Bonferroni-corrected *p* value = 0.038; 95% CI: 0.608–0.903). Furthermore, rs3027178 was associated with AD also after correction for both sex and age of the participants (nominal *p* value = 0.032; 95% CI: 0.617–0.978). Comparable results were obtained when a model not adjusted for sex was used (data not shown).

**Table 3:** Results of logistic regression analysis of the selected SNPs in the validation cohort

*SNP*	*Gene symbol*	*Minor allele*	AD vs CTRL		CENT vs CTRL		AD vs CENT	
			*OR (95% CI)*	*p value*	*OR (95% CI)*	*p value*	*OR (95% CI)*	*p value*
rs3828057	LINGO4;RORC	T	0.857 (0.692–1.061)	0.157	0.848 (0.634–1.134)	0.266	1.009 (0.77–1.324)	0.947
rs1047354	TMEM165;CLOCK	G	0.975 (0.786–1.21)	0.819	0.993 (0.746–1.321)	0.962	1 (0.76–1.315)	0.999
rs62303689	TMEM165;CLOCK	A	1.165 (0.847–1.602)	0.348	1.068 (0.688–1.659)	0.77	1.079 (0.723–1.61)	0.709
rs6828570	TMEM165;CLOCK	G	0.962 (0.779–1.187)	0.718	0.987 (0.747–1.305)	0.927	0.993 (0.76–1.298)	0.96
rs851010	MAPK14	A	0.945 (0.764–1.167)	0.597	0.945 (0.709–1.26)	0.699	0.98 (0.748–1.284)	0.882
rs9470219	MAPK14	A	0.919 (0.745–1.135)	0.434	0.916 (0.688–1.22)	0.549	0.984 (0.752–1.288)	0.906
rs3818559	RORB	C	0.844 (0.678–1.051)	0.13	1.01 (0.756–1.351)	0.946	0.838 (0.629–1.118)	0.229
rs2675703	OPN4	T	1.061 (0.801–1.407)	0.679	1.135 (0.784–1.642)	0.503	0.897 (0.637–1.263)	0.534
rs2254051	OPN4	A	1.063 (0.802–1.408)	0.67	1.111 (0.767–1.609)	0.578	0.922 (0.654–1.299)	0.643
rs3027178	PER1;AC129492.3	G	**0.803 (0.647**–**0.996)**	**0.046**	**0.727 (0.539**–**0.982)**	**0.038**	1.094 (0.827–1.448)	0.53
rs3746682	PROKR2	C	1.083 (0.856–1.368)	0.507	0.725 (0.517–1.016)	0.062	**1.454 (1.056**–**2.002)**	**0.022**
rs8116897	PROKR2	T	1.053 (0.855–1.295)	0.629	1.305 (0.989–1.724)	0.06	0.847 (0.653–1.098)	0.21
rs6008259	PPARA	A	0.962 (0.739–1.254)	0.776	0.968 (0.679–1.38)	0.857	0.995 (0.714–1.387)	0.977

*OR* odds ratio, *CI* 95% confidence interval

Statistically significant results (*p* value < 0.05) are highlighted in bold

**Table 4:** Results of logistic regression analysis of the selected SNPs, combining discovery and validation cohorts

*SNP*	*Gene symbol*	*Minor allele*	AD vs CTRL		CENT vs CTRL		AD vs CENT	
			*OR (95% CI)*	*p value*	*OR (95% CI)*	*p value*	*OR (95% CI)*	*p value*
rs3828057	LINGO4;RORC	T	0.786 (0.646–0.955)	0.015	0.843 (0.64–1.112)	0.226	0.963 (0.737–1.258)	0.78
rs1047354	TMEM165;CLOCK	G	0.888 (0.73–1.079)	0.231	0.925 (0.702–1.218)	0.578	0.996 (0.762–1.301)	0.976
rs62303689	TMEM165;CLOCK	A	1.29 (0.962–1.729)	0.089	1.155 (0.753–1.772)	0.509	1.091 (0.739–1.612)	0.661
rs6828570	TMEM165;CLOCK	G	0.878 (0.725–1.064)	0.184	0.922 (0.703–1.209)	0.557	0.987 (0.759–1.282)	0.919
rs851010	MAPK14	A	1.062 (0.876–1.286)	0.542	1.01 (0.767–1.329)	0.944	1.013 (0.777–1.32)	0.926
rs9470219	MAPK14	A	1.047 (0.865–1.268)	0.637	0.993 (0.756–1.304)	0.957	1.015 (0.779–1.323)	0.912
rs3818559	RORB	C	0.952 (0.779–1.165)	0.635	1.102 (0.832–1.46)	0.498	0.87 (0.656–1.152)	0.331
rs2675703	OPN4	T	0.883 (0.685–1.139)	0.339	1.11 (0.783–1.573)	0.558	0.823 (0.59–1.149)	0.253
rs2254051	OPN4	A	0.876 (0.68–1.129)	0.308	1.087 (0.766–1.543)	0.641	0.839 (0.601–1.172)	0.303
rs3027178	PER1;AC129492.3	G	**0.741 (0.608**–**0.903)**	**0.003**	**0.704 (0.527**–**0.939)**	**0.017**	1.062 (0.806–1.4)	0.67
rs3746682	PROKR2	C	0.918 (0.744–1.133)	0.425	**0.687 (0.497**–**0.95)**	**0.023**	1.346 (0.986–1.837)	0.061
rs8116897	PROKR2	T	1.172 (0.97–1.416)	0.1	**1.385 (1.058**–**1.813)**	**0.018**	0.875 (0.679–1.128)	0.304
rs6008259	PPARA	A	1.127 (0.882–1.44)	0.341	1.067 (0.757–1.504)	0.712	1.029 (0.742–1.427)	0.866

*OR* odds ratio, *CI* 95% confidence interval

Statistically significant results (*p* value < 0.05) are highlighted in bold

Interestingly, we found that rs3027178 was nominally significant also when considering the comparison between CENT and CTRL (*p* value = 0.038), with a direction of the odds ratio analogous to what observed in the AD vs CTRL comparison (OR: 0.727; 95% CI: 0.539 ± 0.982) (Tables 3 and 4). When considering the comparison AD vs CENT (extreme phenotypes), we identified 1 significant SNP (rs3746682; nominal *p* value <0.05), that however did not show a differential trend between AD and CTRL (Tables 3 and 4).

**Fig. 2:**
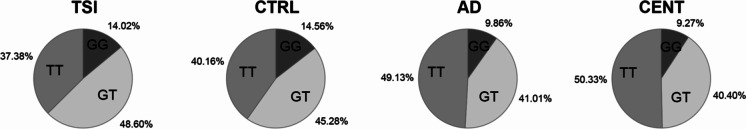
Comparison of the genotypic frequencies of the SNP rs3027178 between the groups under investigation. TSI: Tuscan population from the 1000 genomes

Figure 2 shows the genotypic frequencies of rs3027178 in our entire cohort (combining DC1 and validation samples). The AD group has a frequency distribution similar to CENT, while CTRL distribution resembles the one observed in the Tuscan population from the 1000 genomes (TSI) used as reference population.

### Functional annotation of rs3027178

Finally, we interrogated GTEx portal to investigate possible functional consequences of rs3027178 variability in AD and longevity. We found that rs3027178 is an expression quantitative trait locus (eQTL) for 4 genes on chromosome 17 (*CTC1*, *TMEM107*, *VAMP2* and MIR6883, which maps within *PER1* gene) in a number of tissues (Table s3). Furthermore, rs3027178 is a splicing quantitative trait locus (sQTL) of PER1 and CTC1 in several tissues (Table s4).

## Discussion

This study aimed to investigate the genetic variability of circadian clock genes, including the melanopsin (*OPN4*) gene, in patients with AD compared to cognitively normal controls from the Italian population. We combined a discovery phase based on NGS analysis and a validation phase based on targeted genotyping. In the validation phase, the design of our study also included the comparison with a cohort of centenarians. Centenarians delayed or escaped the major age-related diseases, including AD [59], and can therefore be used as “super-controls” to maximize the phenotypic differences among the groups under study [42].

Our results show that rs3027178, a synonymous variant of *PER1* gene, is associated with AD in the Italian population. We report that the allele rs3027178-G decreases the risk for AD but at the same time also decreases the chance to become centenarian.

While a growing number of evidences support a role of circadian rhythms in AD, only a few studies have specifically investigated the association of polymorphisms in circadian genes with AD so far. SNPs in *BMAL1* and *CLOCK* genes were shown to be associated with susceptibility to AD [60–63]. More recently, Bessi and colleagues reported that *CLOCK* T3111C polymorphism interacts with cardiovascular risk factors in individuals with subjective and mild cognitive impairment, influencing the risk of conversion to AD [64]. Interestingly, SNPs in *CLOCK* gene modulate also aging quality, evaluated according to a series of biochemical, neuropsychological and sleep-related parameters [65].

To the best of our knowledge, the rs3027178 has not been studied in relation to AD so far, particularly in the Italian population. Although the association of rs3027178 with AD was only nominally significant in the discovery phase, it survived multiple testing correction when combining the discovery and the validation cohorts. Furthermore, the association was also significant when correcting for age, suggesting that the genotypic frequencies of this polymorphism are not related to mortality in our cohort.

The fact that AD and centenarians have comparable genotypic frequencies of rs3027178 is only apparently surprising. Indeed, other studies have already reported SNPs associated with both AD and longevity [66–68]. More generally, although centenarians are an excellent model to investigate genetic variants associated with longevity [69], several studies have shown that some gene variants associated with higher risk for various diseases are also present in the genomes of very long-lived people without compromising their health [70–77]. Additionally, it has been reported that conserved pathways of aging simultaneously influence multiple age-related diseases in humans [78].

This apparent paradox may be due to the fact that many genetic variants have a pleiotropic effect, and therefore they can be protective for some diseases but at the same time increase the risk of others. Furthermore, consistent with the notion of antagonistic pleiotropy, the effect of some gene variants changes with age (for example, increasing the risk in the first decades of life, while being protective in old age) and with exposure to environmental factors. Healthy dietary patterns such as Mediterranean Diet can indeed improve health status in older adults [79–83] also reducing the adverse effect of genetic risk variants [84]. Consequently, some risk gene variants may become pro-longevity according to the context [69, 85]. In particular, the cohort of centenarians analyzed in this study also include thirty 105+-year-old healthy individuals who further support the hypothesis that genetic background and lifestyle factors combined together could modulate the expression of specific gene variants causing a protective rather than a risk effect.

Based on these considerations, we reviewed the literature to evaluate the association of the rs3027178 polymorphism with other pathologies. Some studies have reported an association of this variant with different forms of cancer, an interesting observation considering the inverse relationship between tumors and neurodegenerative diseases [86]. However, the observed effect varies depending on the tumor. In some studies, the minor G allele was found to be protective for tumors such as glioma [87], liposarcoma [88] and breast cancer [89], while in other studies it was found to be at risk factor, as in the case of prostate cancer [90] and hepatocellular carcinoma [91]. Conflicting data are reported in gastric cancer [92, 93].

Interestingly, rs3027178 polymorphism can influence the expression of genes that can be relevant for AD, including *VAMP2* in hypothalamus and *CTC1* across several tissues. *VAMP2* encodes for the “vesicle-associated membrane protein 2”, a member of N-ethylmaleimide-sensitive factor attachment protein receptor (SNARE) family. SNAREs are involved in neurotransmitter release, and several reports showed that their expression and activity are deregulated in neurodegenerative diseases [94]. *CTC1* encodes for the “CST Telomere Replication Complex Component 1” protein, which plays an essential role in protecting telomeres from degradation. *CTC1* gene is the target of a non-coding RNA differentially expressed in AD brains [95].

Previous GWAS studies identified some loci showing sex-specific associations with longevity [96, 97]. In our analysis, the adjusted and the unadjusted models returned comparable results, suggesting that the association of rs3027178 with AD and with longevity is not dependent on sex.

Overall, we found a significant association between a SNP located in a relevant circadian gene (*PER1*) and AD in the Italian population. This result underlines the relevance of the potential impact of circadian dysfunction in the predisposition to Alzheimer’s type dementia [24]. The major weakness of this study is represented by the relatively small sample size of the studied cohorts and by the fact that we mostly considered nominally significant *p* values. On the other side, the strength is that this is the first study in which circadian genes have been comprehensively investigated in AD, combining NGS and targeted genotyping approaches and including centenarians in the study design. Further studies on larger and geographically distinct cohorts should evaluate the rs3027178 polymorphism in *PER1* gene in AD and its possible contribution to neurodegeneration.

## CONSORTIUM NAME

*** Italian Multicentric Group on clock genes and actigraphy in AD***: Federico Cucchiara^a,b^, Alessandro Schirru^c^, Lo Gerfo Annalisa^c^, Lombardi Gemma^d^, Arnaldi Dario^e,f^, Pietro Mattioli ^e,f^, Flavio Nobili^e,f^, Gianluigi Cerroni^g.h^ Antonella Bartoli^g,h^, Raffaele Manni^i^, Elena Sinforiani^j^, Michele Terzaghi^i,k^, Maria Grazia Arena^l^, Rosalia Silvestri^m^, Maria Caterina Di Perri^m^, Ferdinando Franzoni^c^, Gloria Tognoni^c^, Michelangelo Mancuso^c^, Sandro Sorbi^n^ , Ubaldo Bonuccelli^c^, Ugo Faraguna^a,o^

## Consortium affiliations:

a: SONNOLab, Department of Translational Research and of New Surgical and Medical Technologies, University of Pisa, Pisa, Italy;

b: Clinical Pharmacology and Pharmacogenetic Unit, Department of Clinical and Experimental Medicine, University of Pisa, Pisa, Italy;

c: Neurology Unit, Department of Clinical and Experimental Medicine, University of Pisa, Pisa, Italy;

d: IRCCS Fondazione Don Carlo Gnocchi, Florence, Italy;

e: Clinical Neurology, Department of Neuroscience (DINOGMI), University of Genoa, Genoa, Italy;

f: IRCCS Ospedale San Martino, Genoa, Italy;

g: Center of Sleep Medicine, Villa Serena Hospital, Città S. Angelo, Pescara, Italy;

h: Villaserena Foundation for the Research, Città S. Angelo, Pescara, Italy;

i: Sleep and Epilepsy Unit, IRCCS Mondino Foundation, Pavia, Italy;

j: Neuropsychology/Alzheimer’s Disease Assessment Unit, IRCCS Mondino Foundation, Pavia, Italy;

k: Department of Brain and Behavioural Sciences, University of Pavia, Pavia, Italy;

l: Center for Cognitive Disorders and Dementias, Alzheimer’s Disease Assessment Unit, UOC of Neurology and Neuromuscular Disorders, AOU Policlinico, “G. Martino”, University of Messina, Messina, Italy;

m: Sleep Medicine Center, UOSD of Neurophysiopathology and Movement Disorders, AOU Policlinico “G.~Martino”, Department of Clinical and Experimental Medicine, University of Messina, Italy;

n: IRCCS Fondazione Don Carlo Gnocchi, Florence, Italy; Department of Neuroscience, Psychology, Drug Research and Child Health, University of Florence, Florence, Italy;

o: Department of Developmental Neuroscience, IRCCS Stella Maris Foundation, Pisa, Italy

## Supplementary Information

Below is the link to the electronic supplementary material.

Supplementary file1 (JPG 81 KB)

Supplementary file2 (DOCX 25 KB)

Supplementary file3 (XLSX 156 KB)

## References

[1] Schroeder AM, Colwell CS (2013). How to fix a broken clock. Trends Pharmacol Sci.

[2] Ruan W, Yuan X, Eltzschig HK (2021). Circadian rhythm as a therapeutic target. Nat Rev Drug Discov.

[3] Buhr ED, Yoo S-H, Takahashi JS (2010). Temperature as a universal resetting cue for mammalian circadian oscillators. Science.

[4] Takahashi JS (2017). Transcriptional architecture of the mammalian circadian clock. Nat Rev Genet.

[5] Vitaterna M, King D, Chang A (1994). Mutagenesis and mapping of a mouse gene, Clock, essential for circadian behavior. Science.

[6] Ko CH, Takahashi JS (2006). Molecular components of the mammalian circadian clock. Hum Mol Genet.

[7] Bunger MK, Wilsbacher LD, Moran SM (2000). Mop3 is an essential component of the master circadian pacemaker in mammals. Cell.

[8] Rijo-Ferreira F, Takahashi JS (2019). Genomics of circadian rhythms in health and disease. Genome Med.

[9] Berson DM (2002). Phototransduction by retinal ganglion cells that set the circadian clock. Science.

[10] Hattar S (2002). Melanopsin-containing retinal ganglion cells: architecture, projections, and intrinsic photosensitivity. Science.

[11] Hannibal J (2002). Neurotransmitters of the retino-hypothalamic tract. Cell Tissue Res.

[12] Aranda ML, Schmidt TM (2021). Diversity of intrinsically photosensitive retinal ganglion cells: circuits and functions. Cell Mol Life Sci.

[13] Roecklein KA, Rohan KJ, Duncan WC (2009). A missense variant (P10L) of the melanopsin (OPN4) gene in seasonal affective disorder. J Affect Disord.

[14] Higuchi S, Hida A, Tsujimura S (2013). Melanopsin gene polymorphism I394T is associated with pupillary light responses in a dose-dependent manner. PLoS ONE.

[15] Lee S, Hida A, Kitamura S (2014). Association between the melanopsin gene polymorphism OPN4*Ile394Thr and sleep/wake timing in Japanese university students. J Physiol Anthropol.

[16] Rodgers J, Hughes S, Pothecary CA (2018). Defining the impact of melanopsin missense polymorphisms using in vivo functional rescue. Hum Mol Genet.

[17] Rodgers J, Peirson SN, Hughes S, Hankins MW (2018). Functional characterisation of naturally occurring mutations in human melanopsin. Cell Mol Life Sci.

[18] Logan RW, McClung CA (2019). Rhythms of life: circadian disruption and brain disorders across the lifespan. Nat Rev Neurosci.

[19] Fifel K, Videnovic A (2021) Circadian and sleep dysfunctions in neurodegenerative disorders—an update. Front Neurosci 14.10.3389/fnins.2020.627330 10.3389/fnins.2020.627330 PMC7848154 33536872

[20] Vitiello MV, Prinz PN, Williams DE (1990). Sleep disturbances in patients with mild-stage Alzheimer’s disease. J Gerontol.

[21] Rothman SM, Mattson MP (2012). Sleep disturbances in Alzheimer’s and Parkinson’s diseases. Neuromol Med.

[22] Musiek ES, Bhimasani M, Zangrilli MA (2018). Circadian rest-activity pattern changes in aging and preclinical Alzheimer disease. JAMA Neurol.

[23] Leng Y, Musiek ES, Hu K (2019). Association between circadian rhythms and neurodegenerative diseases. Lancet Neurol.

[24] Tranah GJ, Blackwell T, Stone KL (2011). Circadian activity rhythms and risk of incident dementia and mild cognitive impairment in older women. Ann Neurol.

[25] Özcan GG, Lim S, Leighton PL, et al (2020) Sleep is bi-directionally modified by amyloid beta oligomers. eLife 9. 10.7554/eLife.53995 10.7554/eLife.53995 PMC7360368 32660691

[26] Wu Y-H, Swaab DF (2007). Disturbance and strategies for reactivation of the circadian rhythm system in aging and Alzheimer’s disease. Sleep Med.

[27] Oosterman JM, van Someren EJW, Vogels RLC (2009). Fragmentation of the rest-activity rhythm correlates with age-related cognitive deficits. J Sleep Res.

[28] Froy O (2011). Circadian rhythms, aging, and life span in mammals. Physiology.

[29] Duncan MJ (2020). Interacting influences of aging and Alzheimer’s disease on circadian rhythms. Eur J Neurosci.

[30] Franceschi C, Garagnani P, Morsiani C (2018). The continuum of aging and age-related diseases: common mechanisms but different rates. Front Med (Lausanne).

[31] Johnson BM, Miao M, Sadun AA (1987). Age-related decline of human optic nerve axon populations. Age.

[32] Feuer WJ, Budenz DL, Anderson DR (2011). Topographic differences in the age-related changes in the retinal nerve fiber layer of normal eyes measured by stratus optical coherence tomography. J Glaucoma.

[33] La Morgia C, Ross-Cisneros FN, Koronyo Y (2016). Melanopsin retinal ganglion cell loss in Alzheimer disease. Ann Neurol.

[34] Zhang L, Ptáček LJ, Fu Y-H. Diversity of human clock genotypes and consequences. Prog Mol Biol Transl Sci. 2013;119:51–81. 10.1016/B978-0-12-396971-2.00003-8. 10.1016/B978-0-12-396971-2.00003-8 PMC4169291 23899594

[35] Franceschi C, Passarino G, Mari D, Monti D (2017). Centenarians as a 21st century healthy aging model: a legacy of humanity and the need for a world-wide consortium (WWC100+). Mech Ageing Dev.

[36] Franceschi C, Ostan R, Santoro A (2018). Nutrition and inflammation: are centenarians similar to individuals on calorie-restricted diets?. Ann Rev Nutr.

[37] Santoro A, Martucci M, Conte M (2020). Inflammaging, hormesis and the rationale for anti-aging strategies. Ageing Res Rev.

[38] Santoro A, Ostan R, Candela M (2018). Gut microbiota changes in the extreme decades of human life: a focus on centenarians. Cell Mol Life Sci.

[39] Santoro A, Zhao J, Wu L (2020). Microbiomes other than the gut: inflammaging and age-related diseases. Sem Immunopathol.

[40] Tafaro L, Cicconetti P, Baratta A (2007). Sleep quality of centenarians: cognitive and survival implications. Arch Gerontol Geriatr.

[41] Garagnani P, Marquis J, Delledonne M, et al (2021) Whole-genome sequencing analysis of semi-supercentenarians. eLife 10. 10.7554/eLife.57849 10.7554/eLife.57849 PMC8096429 33941312

[42] Garagnani P, Giuliani C, Pirazzini C (2013). Centenarians as super-controls to assess the biological relevance of genetic risk factors for common age-related diseases: a proof of principle on type 2 diabetes. Aging (Albany NY).

[43] Guarnieri B, Maestri M, Cucchiara F (2020). Multicenter study on sleep and circadian alterations as objective markers of mild cognitive impairment and Alzheimer’s disease reveals sex differences. J Alzheimer’s Dis.

[44] Dubois B, Feldman HH, Jacova C (2014). Advancing research diagnostic criteria for Alzheimer’s disease: the IWG-2 criteria. Lancet Neurol.

[45] McKhann G, Drachman D, Folstein M (1984). Clinical diagnosis of Alzheimer’s disease: report of the NINCDS-ADRDA Work Group under the auspices of Department of Health and Human Services Task Force on Alzheimer’s Disease. Neurology.

[46] Petersen RC (2004). Mild cognitive impairment as a diagnostic entity. J Intern Med.

[47] Gallassi R, Lenzi P, Stracciari A (1986). Neuropsychological assessment of mental deterioration: purpose of a brief battery and a probabilistic definition of “normality” and “non-normality”. Acta Psychiatr Scand.

[48] Gallassi R, Oppi F, Poda R (2010). Are subjective cognitive complaints a risk factor for dementia?. Neurol Sci.

[49] Arosio B, Ostan R, Mari D (2017). Cognitive status in the oldest old and centenarians: a condition crucial for quality of life methodologically difficult to assess. Mech Ageing Dev.

[50] Li H, Durbin R (2009). Fast and accurate short read alignment with Burrows-Wheeler transform. Bioinformatics.

[51] DePristo MA, Banks E, Poplin R (2011). A framework for variation discovery and genotyping using next-generation DNA sequencing data. Nat Genet.

[52] Li H, Handsaker B, Wysoker A (2009). The Sequence Alignment/Map format and SAMtools. Bioinformatics.

[53] Cingolani P, Platts A, Wang LL (2012). A program for annotating and predicting the effects of single nucleotide polymorphisms, SnpEff. Fly.

[54] Danecek P, Auton A, Abecasis G (2011). The variant call format and VCFtools. Bioinformatics.

[55] Purcell S, Neale B, Todd-Brown K (2007). PLINK: a tool set for whole-genome association and population-based linkage analyses. Am J Hum Genet.

[56] Pippucci T, Licchetta L, Baldassari S (2019). Contribution of ultrarare variants in mTOR pathway genes to sporadic focal epilepsies. Ann Clin Transl Neurol.

[57] Mehrotra DV, Chan ISF, Berger RL (2003). A cautionary note on exact unconditional inference for a difference between two independent binomial proportions. Biometrics.

[58] Gabriel S, Ziaugra L, Tabbaa D (2009) SNP genotyping using the SequenomMassARRAY iPLEX platform. Current Protocols in Human Genetics 60.10.1002/0471142905.hg0212s60 10.1002/0471142905.hg0212s60 19170031

[59] Giuliani C, Pirazzini C, Delledonne M (2017). Centenarians as extreme phenotypes: an ecological perspective to get insight into the relationship between the genetics of longevity and age-associated diseases. Mech Ageing Dev.

[60] Chen H, Huang C, You C (2013). Polymorphism of CLOCK gene rs 4580704 C>G is associated with susceptibility of Alzheimer’s disease in a Chinese population. Arch Med Res.

[61] Chen Q, Huang C-Q, Hu X-Y (2013). Functional CLOCK gene rs1554483 G/C polymorphism is associated with susceptibility to Alzheimer’s disease in the Chinese population. J Int Med Res.

[62] Hastings MH, Goedert M (2013). Circadian clocks and neurodegenerative diseases: time to aggregate?. Curr Opin Neurobiol.

[63] Yang Y-K, Peng X-D, Li Y-H (2013). The polymorphism of CLOCK gene 3111T/C C>T Is associated with susceptibility of Alzheimer disease in Chinese population. J Investig Med.

[64] Bessi V, Balestrini J, Bagnoli S (2020). Influence of ApoE genotype and clock T3111C interaction with cardiovascular risk factors on the progression to Alzheimer’s disease in subjective cognitive decline and mild cognitive impairment patients. J Personalized Med.

[65] Pagliai G, Sofi F, Dinu M (2019). CLOCK gene polymorphisms and quality of aging in a cohort of nonagenarians—the MUGELLO study. Sci Rep.

[66] Crocco P, Saiardi A, Wilson MS, et al (2016) Contribution of polymorphic variation of inositol hexakisphosphate kinase 3 (IP6K3) gene promoter to the susceptibility to late onset Alzheimer’s disease. Biochim Biophys Acta (BBA) – Mol Basis Dis 1862:1766–1773. 10.1016/j.bbadis.2016.06.014 10.1016/j.bbadis.2016.06.014 27345265

[67] De Rango F, Crocco P, Iannone F (2019). Inositol polyphosphate multikinase (IPMK), a gene coding for a potential moonlighting protein, contributes to human female longevity. Genes.

[68] Dato S, Crocco P, De Rango F (2021). IP6K3 and IPMK variations in LOAD and longevity: evidence for a multifaceted signaling network at the crossroad between neurodegeneration and survival. Mech Ageing Dev.

[69] Franceschi C, Garagnani P, Olivieri F (2020). The contextualized genetics of human longevity. J Am Coll Cardiol.

[70] Freudenberg-Hua Y, Freudenberg J, Vacic V (2014). Disease variants in genomes of 44 centenarians. Mol Genet Genomic Med.

[71] Beekman M, Blanché H, Perola M (2013). Genome-wide linkage analysis for human longevity: genetics of healthy aging study. Aging Cell.

[72] Deelen J, Beekman M, Uh H-W (2014). Genome-wide association meta-analysis of human longevity identifies a novel locus conferring survival beyond 90 years of age. Hum Mol Genet.

[73] Lescai F, Chiamenti AM, Codemo A (2011). An APOE haplotype associated with decreased ε4 expression increases the risk of late onset Alzheimer’s disease. J Alzheimer’s Dis.

[74] Naj AC, Jun G, Reitz C (2014). Effects of multiple genetic loci on age at onset in late-onset Alzheimer disease. JAMA Neurol.

[75] Wang X, Lopez OL, Sweet RA (2014). Genetic determinants of disease progression in Alzheimer’s disease. J Alzheimer’s Dis.

[76] Maxwell TJ, Ballantyne CM, Cheverud JM (2013). APOE modulates the correlation between triglycerides, cholesterol, and CHD through pleiotropy, and gene-by-gene interactions. Genetics.

[77] Hellwege JN, Palmer ND, Raffield LM (2014). Genome-wide family-based linkage analysis of exome chip variants and cardiometabolic risk. Genet Epidemiol.

[78] Johnson SC, Dong X, Vijg J, Suh Y (2015). Genetic evidence for common pathways in human age-related diseases. Aging Cell.

[79] Marseglia A, Xu W, Fratiglioni L, et al (2018) Effect of the NU-AGE diet on cognitive functioning in older adults: a randomized controlled trial. Front Physiol 9.10.3389/fphys.2018.00349 10.3389/fphys.2018.00349 PMC5893841 29670545

[80] Bonaccio M, Di Castelnuovo A, Costanzo S (2018). Mediterranean diet and mortality in the elderly: a prospective cohort study and a meta-analysis. Br J Nutr.

[81] Jennings A, Berendsen AM, de Groot LCPGM (2019). Mediterranean-style diet improves systolic blood pressure and arterial stiffness in older adults. Hypertension.

[82] Gensous N, Garagnani P, Santoro A, et al (2020) One-year Mediterranean diet promotes epigenetic rejuvenation with country- and sex-specific effects: a pilot study from the NU-AGE project. Geroscience. 10.1007/s11357-019-00149-0 10.1007/s11357-019-00149-0 PMC7205853 31981007

[83] Ghosh TS, Rampelli S, Jeffery IB (2020). Mediterranean diet intervention alters the gut microbiome in older people reducing frailty and improving health status: the NU-AGE 1-year dietary intervention across five European countries. Gut.

[84] Corella D, Carrasco P, Sorli JV (2013). Mediterranean diet reduces the adverse effect of the TCF7L2-rs7903146 polymorphism on cardiovascular risk factors and stroke incidence: a randomized controlled trial in a high-cardiovascular-risk population. Diabetes Care.

[85] Ukraintseva S, Yashin A, Arbeev K (2016). Puzzling role of genetic risk factors in human longevity: “risk alleles” as pro-longevity variants. Biogerontology.

[86] Driver JA (2014). Inverse association between cancer and neurodegenerative disease: review of the epidemiologic and biological evidence. Biogerontology.

[87] Madden MH, Anic GM, Thompson RC (2014). Circadian pathway genes in relation to glioma risk and outcome. Cancer Causes Control.

[88] Benna C, Rajendran S, Spiro G (2018). Associations of clock genes polymorphisms with soft tissue sarcoma susceptibility and prognosis. J Transl Med.

[89] Lesicka M, Jabłońska E, Wieczorek E (2019). Circadian gene polymorphisms associated with breast cancer susceptibility. Int J Mol Sci.

[90] Zhu Y, Stevens RG, Hoffman AE (2009). Testing the circadian gene hypothesis in prostate cancer: a population-based case-control study. Cancer Res.

[91] Zhang Z, Ma F, Zhou F (2014). Functional polymorphisms of circadian negative feedback regulation genes are associated with clinical outcome in hepatocellular carcinoma patients receiving radical resection. Med Oncol.

[92] Qu F, Qiao Q, Wang N (2016). Genetic polymorphisms in circadian negative feedback regulation genes predict overall survival and response to chemotherapy in gastric cancer patients. Sci Rep.

[93] Rajendran S, Benna C, Marchet A (2020). Germline polymorphisms of circadian genes and gastric cancer predisposition. Cancer Commun.

[94] Margiotta A (2021). Role of SNAREs in neurodegenerative diseases. Cells.

[95] Qiu W, Guo X, Lin X (2017). Transcriptome-wide piRNA profiling in human brains of Alzheimer’s disease. Neurobiol Aging.

[96] Zeng Y, Nie C, Min J (2018). Sex differences in genetic associations with longevity. JAMA Netw Open.

[97] Seripa D, Franceschi M, Matera MG (2006). Sex differences in the association of apolipoprotein E and angiotensin-converting enzyme gene polymorphisms with healthy aging and longevity: a population-based study from Southern Italy. J Gerontol A Biol Sci Med Sci.

