# Supplementary figures and images for: Association of rs3027178 polymorphism in the circadian clock gene PER1 with susceptibility to Alzheimer’s disease and longevity in an Italian population
Source: GeroScience. 2021 Dec 18;44(2):881–96. doi: 10.1007/s11357-021-00477-0 (PMC9135916; doi:10.1007/s11357-021-00477-0)

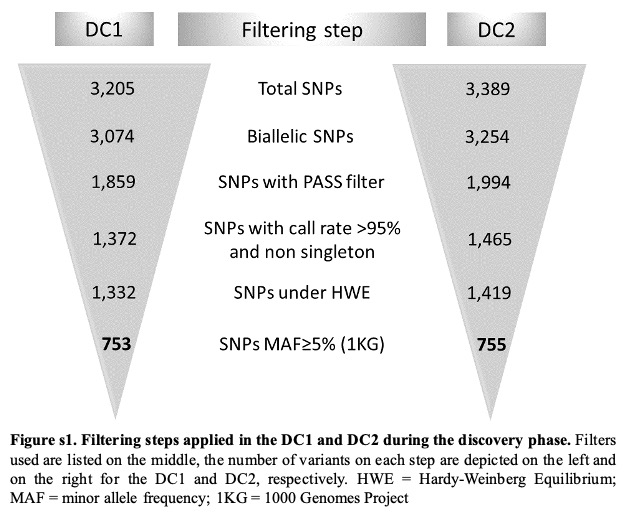

Supplement: Supplementary file 1 — Supplementary file1 (JPG 81 KB) [file 11357_2021_477_MOESM1_ESM.jpg]
