# Supplementary material for: Association of rs3027178 polymorphism in the circadian clock gene PER1 with susceptibility to Alzheimer’s disease and longevity in an Italian population
Source: GeroScience. 2021 Dec 18;44(2):881–96. doi: 10.1007/s11357-021-00477-0 (PMC9135916; doi:10.1007/s11357-021-00477-0)
Supplement: Supplementary file 2 — Supplementary file2 (DOCX 25 KB) [file 11357_2021_477_MOESM2_ESM.docx]

**Table s1. List of the 84 genes included in the NGS panel analyzed in the discovery phase.**

| ***FUNCTION*** | ***GENES*** |
| --- | --- |
| Circadian Clock | *ARNTL (BMAL1), ARNTL2, BHLHE40 (DEC1), BHLHE41, CLOCK, CSNK1E, CRY1, CRY2, NR1D1, NR1D2 (REV-ERB), PER1, PER2, PER3, RORA, TIMELESS* |
| Casein Kinases | *CSNK1A1, CSNK1D, CSNK1E, CSNK2A1, CSNK2A2* |
| CREB Signaling | *AANAT, CAMK2A, CAMK2B, CAMK2D, CAMK2G, CHRNB2, CREB1, CREB3, KCNMA1, HTR7, MAPK1, MAPK14, MAPK3, MAT2A, PRKACB, PRKACG, PRKAR1A, PRKAR1B, PRKAR2A, PRKAR2B, PRKCA, PRKCB, PROKR2* |
| Melatonin Receptors | *MTNR1A, MTNR1B* |
| Opsins | *OPN3, OPN4* |
| Circadian Regulated Transcription Factors | *ALAS1, EGR1, EGR3, EPO, ESRRA, HLF, IRF1, MYOD1, NFIL3, NKX2-5, PAX4, POU2F1, RORB, RORC, SMAD4, SP1, SREBF1, STAT5A, TEF, TFAP2A, TGFB1, WEE1, DBP, PPARA* |
| Other Common Circadian Regulated Genes | *CARTPT, CCRN4L, FBXL21, FBXL3, HEBP1, NCOA3, NMS, NPAS2, NR2F6, PPARGC1A, PRF1, PTGDS, SLC9A3* |
| Others | *CHRNB2, CRX* |

**Table s2: List of the SNPs analyzed in the validation phase.**

| **SNP** | **Chromosome** | **Position** | **Symbol** | **Discovery cohort** | **Quality check** |
| --- | --- | --- | --- | --- | --- |
| rs3828057 | chr1 | 151807701 | LINGO4;RORC | DC1 and DC2 | X |
| rs1047354 | chr4 | 55429416 | TMEM165;CLOCK | DC2 | X |
| rs62303689 | chr4 | 55429706 | TMEM165;CLOCK | DC2 | X |
| rs6828570 | chr4 | 55431595 | TMEM165;CLOCK | DC2 | X |
| rs851010 | chr6 | 36073654 | MAPK14 | DC1 and DC2 | X |
| rs9470219 | chr6 | 36107442 | MAPK14 | DC1 and DC2 | X |
| rs3818559 | chr9 | 74671960 | RORB | DC1 and DC2 | X |
| rs2675703 | chr10 | 86654812 | OPN4 | DC1 and DC2 | X |
| rs2254051 | chr10 | 86655001 | OPN4 | DC1 and DC2 | X |
| rs3027178 | chr17 | 8149767 | PER1;AC129492.3 | DC1 | X |
| rs3746682 | chr20 | 5302610 | PROKR2 | DC1 and DC2 | X |
| rs8116897 | chr20 | 5313850 | PROKR2 | DC1 | X |
| rs1134224 | chr22 | 46633371 | PPARA | DC1 and DC2 |  |
| rs6008259 | chr22 | 46237885 | PPARA | DC1 and DC2 | X |

SNP: name of the single nucleotide polymorphism; Chromosome: chromosomal location; Position: coordinates of the SNP in the chromosome, according to GRCh37/hg19 assembly; Symbol: name of the gene; Discovery cohort: the column indicates whether the SNP was identified in DC1 (AD vs CTRL), DC2 (AD+MCI vs CTRL) or both; Quality check: the column indicates whether the SNP passed quality control for MassARRAY data.

**Table s3. Significant expression quantitative trait loci (eQTL) for rs3027178**

| Gencode Id | Gene Symbol | P-Value | NES | Tissue |
| --- | --- | --- | --- | --- |
| ENSG00000178971.13 | CTC1 | 0.000051 | -0.11 | Thyroid |
| ENSG00000178971.13 | CTC1 | 0.00003 | -0.12 | Cells - Cultured fibroblasts |
| ENSG00000178971.13 | CTC1 | 0.0000016 | -0.13 | Skin - Sun Exposed (Lower leg) |
| ENSG00000179029.14 | TMEM107 | 0.0000039 | -0.13 | Nerve - Tibial |
| ENSG00000178971.13 | CTC1 | 0.0000077 | -0.14 | Breast - Mammary Tissue |
| ENSG00000179029.14 | TMEM107 | 0.000016 | -0.14 | Skin - Sun Exposed (Lower leg) |
| ENSG00000178971.13 | CTC1 | 0.0000014 | -0.16 | Esophagus - Mucosa |
| ENSG00000178971.13 | CTC1 | 2.90E-07 | -0.17 | Nerve - Tibial |
| ENSG00000178971.13 | CTC1 | 3.10E-09 | -0.18 | Heart - Left Ventricle |
| ENSG00000178971.13 | CTC1 | 2.00E-10 | -0.19 | Adipose - Visceral (Omentum) |
| ENSG00000284117.1 | MIR6883 | 0.000076 | -0.2 | Adipose - Visceral (Omentum) |
| ENSG00000178971.13 | CTC1 | 1.10E-09 | -0.24 | Small Intestine - Terminal Ileum |
| ENSG00000220205.8 | VAMP2 | 0.000024 | -0.24 | Brain - Hypothalamus |
| ENSG00000178971.13 | CTC1 | 2.90E-18 | -0.26 | Adipose - Subcutaneous |
| ENSG00000178971.13 | CTC1 | 6.50E-43 | -0.27 | Whole Blood |
| ENSG00000178971.13 | CTC1 | 3.30E-13 | -0.3 | Colon - Transverse |
| ENSG00000178971.13 | CTC1 | 5.60E-28 | -0.32 | Lung |
| ENSG00000178971.13 | CTC1 | 2.80E-18 | -0.4 | Spleen |

P-Value: nominal p-value; NES: Normalized Effect Size

**Table s4. Significant splicing quantitative trait loci (sQTL) for rs3027178**

| Gencode Id | Gene Symbol | Intron Id | P-Value | NES | Tissue |
| --- | --- | --- | --- | --- | --- |
| ENSG00000179094.15 | PER1 | 8141340:8141589:clu_12193 | 5.80E-36 | 0.67 | Artery - Tibial |
| ENSG00000179094.15 | PER1 | 8141340:8141589:clu_13323 | 4.40E-23 | 0.47 | Adipose - Subcutaneous |
| ENSG00000179094.15 | PER1 | 8141340:8141589:clu_13903 | 5.30E-22 | 0.47 | Lung |
| ENSG00000179094.15 | PER1 | 8141340:8141589:clu_13159 | 3.00E-20 | 0.43 | Skin - Not Sun Exposed (Suprapubic) |
| ENSG00000179094.15 | PER1 | 8141340:8141589:clu_13159 | 3.00E-20 | 0.43 | Skin - Not Sun Exposed (Suprapubic) |
| ENSG00000179094.15 | PER1 | 8141340:8141805:clu_12915 | 1.70E-19 | -0.52 | Adipose - Visceral (Omentum) |
| ENSG00000179094.15 | PER1 | 8141340:8141805:clu_12915 | 1.70E-19 | -0.52 | Adipose - Visceral (Omentum) |
| ENSG00000179094.15 | PER1 | 8141340:8141589:clu_10498 | 2.70E-19 | 0.41 | Whole Blood |
| ENSG00000179094.15 | PER1 | 8141340:8141589:clu_13744 | 7.10E-19 | 0.51 | Nerve - Tibial |
| ENSG00000179094.15 | PER1 | 8141340:8141589:clu_11785 | 1.80E-18 | 0.46 | Artery - Aorta |
| ENSG00000179094.15 | PER1 | 8141340:8141589:clu_13482 | 4.40E-17 | 0.42 | Skin - Sun Exposed (Lower leg) |
| ENSG00000178971.13 | CTC1 | 8235285:8236083:clu_13342 | 4.10E-16 | -0.37 | Adipose - Subcutaneous |
| ENSG00000179094.15 | PER1 | 8141340:8141589:clu_13287 | 1.40E-15 | 0.43 | Breast - Mammary Tissue |
| ENSG00000178971.13 | CTC1 | 8235285:8236083:clu_12210 | 7.50E-15 | -0.33 | Artery - Tibial |
| ENSG00000179094.15 | PER1 | 8141340:8141589:clu_11633 | 2.50E-14 | 0.4 | Heart - Atrial Appendage |
| ENSG00000179094.15 | PER1 | 8141340:8141589:clu_10631 | 6.10E-14 | 0.6 | Ovary |
| ENSG00000179094.15 | PER1 | 8141340:8141589:clu_14029 | 1.70E-13 | 0.33 | Thyroid |
| ENSG00000179094.15 | PER1 | 8141340:8141589:clu_14029 | 1.70E-13 | 0.33 | Thyroid |
| ENSG00000178971.13 | CTC1 | 8237519:8238392:clu_13176 | 4.80E-12 | -0.36 | Skin - Not Sun Exposed (Suprapubic) |
| ENSG00000178971.13 | CTC1 | 8237519:8238392:clu_13922 | 2.80E-11 | -0.39 | Lung |
| ENSG00000178971.13 | CTC1 | 8237519:8238392:clu_13922 | 2.80E-11 | -0.39 | Lung |
| ENSG00000178971.13 | CTC1 | 8235285:8236083:clu_10622 | 3.30E-11 | -0.61 | Brain - Cerebellar Hemisphere |
| ENSG00000179094.15 | PER1 | 8141340:8141589:clu_23402 | 1.20E-10 | 0.46 | Testis |
| ENSG00000179094.15 | PER1 | 8141340:8141589:clu_23402 | 1.20E-10 | 0.46 | Testis |
| ENSG00000179094.15 | PER1 | 8141340:8141805:clu_11030 | 3.30E-10 | -0.45 | Artery - Coronary |
| ENSG00000179094.15 | PER1 | 8141340:8141805:clu_11880 | 7.50E-10 | -0.41 | Colon - Sigmoid |
| ENSG00000178971.13 | CTC1 | 8235285:8236083:clu_11084 | 8.20E-09 | -0.46 | Brain - Cerebellum |
| ENSG00000179094.15 | PER1 | 8141340:8141805:clu_11861 | 1.10E-08 | -0.23 | Stomach |
| ENSG00000178971.13 | CTC1 | 8237519:8238392:clu_10651 | 1.70E-08 | -0.55 | Ovary |
| ENSG00000178971.13 | CTC1 | 8237519:8238392:clu_12932 | 1.90E-08 | -0.34 | Adipose - Visceral (Omentum) |
| ENSG00000125434.10 | SLC25A35 | 8292355:8292523:clu_23442 | 5.00E-08 | 0.49 | Testis |
| ENSG00000178971.13 | CTC1 | 8237519:8238392:clu_23421 | 1.40E-07 | -0.42 | Testis |
| ENSG00000125434.10 | SLC25A35 | 8292355:8292523:clu_11913 | 4.00E-07 | 0.38 | Colon - Sigmoid |
| ENSG00000178971.13 | CTC1 | 8235285:8236083:clu_11900 | 4.10E-07 | -0.31 | Colon - Sigmoid |
| ENSG00000179094.15 | PER1 | 8141340:8141805:clu_12803 | 4.40E-07 | -0.27 | Colon - Transverse |
| ENSG00000179094.15 | PER1 | 8141340:8141805:clu_12803 | 4.40E-07 | -0.27 | Colon - Transverse |
| ENSG00000179094.15 | PER1 | 8141340:8141805:clu_10594 | 9.90E-07 | -0.58 | Minor Salivary Gland |
| ENSG00000179094.15 | PER1 | 8141340:8141805:clu_10431 | 0.0000012 | -0.51 | Vagina |
